# TITINdb—a computational tool to assess titin’s role as a disease gene

**DOI:** 10.1093/bioinformatics/btx424

**Published:** 2017-07-04

**Authors:** Anna Laddach, Mathias Gautel, Franca Fraternali

**Affiliations:** Randall Division of Cell and Molecular Biophysics, King’s College London BHF Centre of Research Excellence, London, UK

## Abstract

**Summary:**

Large numbers of rare and unique titin missense variants have been discovered in both healthy and disease cohorts, thus the correct classification of variants as pathogenic or non-pathogenic has become imperative. Due to titin’s large size (363 coding exons), current web applications are unable to map titin variants to domain structures. Here, we present a web application, TITINdb, which integrates titin structure, variant, sequence and isoform information, along with pre-computed predictions of the impact of non-synonymous single nucleotide variants, to facilitate the correct classification of titin variants.

**Availability and implementation:**

TITINdb can be freely accessed at http://fraternalilab.kcl.ac.uk/TITINdb

**Supplementary information:**

Supplementary data are available at *Bioinformatics* online.

## 1 Introduction

The giant protein titin, encoded by the gene TTN, is 35 991 amino acids in length [inferred complete (IC) isoform], weighs over 4000 kDa and spans half a sarcomere. Since the advent of next generation sequencing (NGS) technology, a number of titin missense variants, both recessive and dominant, have been associated with disease (both skeletal and cardiac forms of myopathy) (Chauveau *et al.*, 2014a; Hastings *et al.*, 2016; Helle *et al.*, 2016; Savarese *et al.*, 2016), including those which can lead to sudden cardiac death [e.g. hypertrophic cardiomyopathy (HCM)]. Unfortunately, due to titin’s large size, even the majority of healthy individuals possess one or more rare titin missense variants (Lopes *et al.*, 2013). This results in the paradox that rare titin variants are commonly found; therefore, pathogenicity cannot be inferred from frequency alone. To complicate matters further, it has recently been shown that certain titin variants can be pathogenic in particular constellations or act as phenotype modifiers (Evilä *et al.*, 2014). One such scenario is the inheritance of a truncating variant along with a rare or unique missense variant in compound heterozygosity [as has been observed in childhood core myopathy with heart disease, with rare recessive mutations also found in the general population (Chauveau *et al.*, 2014b)]. In light of this information, we believe the assessment of the impact of non-synonymous single nucleotide variants (nsSNVs) at the molecular level to be essential, and propose that *in silico* analyses can be used to prioritise variants for further experimental investigation. We have created TITINdb to facilitate such prioritization.

## 2 Implementation and features

TITINdb includes disease-associated nsSNVs reported in the literature as well as population nsSNVs from the gnomAD database (Lek *et al.*, 2016) and 1000 genomes project (Auton *et al.*, 2015). Additionally, *in silico* saturation mutagenesis has been performed to allow users to access predictions for the impact of any possible single amino acid variants (SAVs). As experimental structures were only available for 23 of titin’s 302 globular domains (132 Fn3, 169 Ig, 1 Kinase), an automated pipeline based on the Modeller software (Webb and Sali, 2016) was set up to model all 279 domains without structure (see Supplementary Figure S3 for more details). As major bioinformatics resources did not agree on titin domain numbers and boundaries, we found it necessary to define these prior to modelling. As illustrated in Supplementary Figure S6, the TITINdb pipeline has greatly increased the structural coverage of titin domains and the quality of the coverage.

Sequence-based prediction of the impact of all nsSNVs was performed using the Condel software (González-Pérez and López-Bigas, 2011). The *in silico* assessment of the impact of nsSNVs using structural information was performed using the DUET software (Pires *et al.*, 2014a) for all known nsSNVs which map to domain structures, additionally the mCSM software (Pires *et al.*, 2014b) was used to predict the impact of all possible SAVs. Where experimental structures of titin domains in complex with binary interaction partners exist, mCSM was also used to predict the impact of SAVs on protein–protein binding affinity. Other structural analysis provided by the application includes computation of the quotient solvent accessible surface area [Q(SASA)] of all residues which map to structure [calculated using POPS (Cavallo *et al.*, 2003)] and predictions of which residues are involved in protein–protein interactions [calculated using SPIDDER (Porollo *et al.*, 2007)]. Of note, however, is the absence of experimental, molecularly resolved protein–protein interaction data for most of titin’s domains, precluding detailed impact analysis on protein–protein interactions. Additionally, nsSNVs are annotated with functional site information from UniProt (The UniProt Consortium, 2017), including residue modifications.

Representative structures for each domain were used in the computation of all structural analyses, apart from the calculation of Q(SASA). This was calculated separately for each structure. A list of structure representatives can be found in the Supplementary Tables S1 and S2.

The application enables users to perform a number of visualizations, which include viewing population nsSNVs as distributions on structures, colour-coded by minor allele frequencies. Additionally, users are able to confidentially upload their own structure for nsSNV visualization (this may be useful if a group has an unpublished crystal structure or believe their own model to be of better quality).

All structures and *in silico* analyses can be freely accessed and downloaded. Additionally, we provide quality assessment of the models (in the form of zDOPE scores and per-residue DOPE plots) (Shen and Sali, 2006) along with the alignments used for homology modelling.

Video tutorials showing the use of TITINdb can be found at http://fraternalilab.kcl.ac.uk/TITINdb/tutorials/

## 3 Applications

### 3.1 Investigating disease associated nsSNVs

**Fig. 1:**
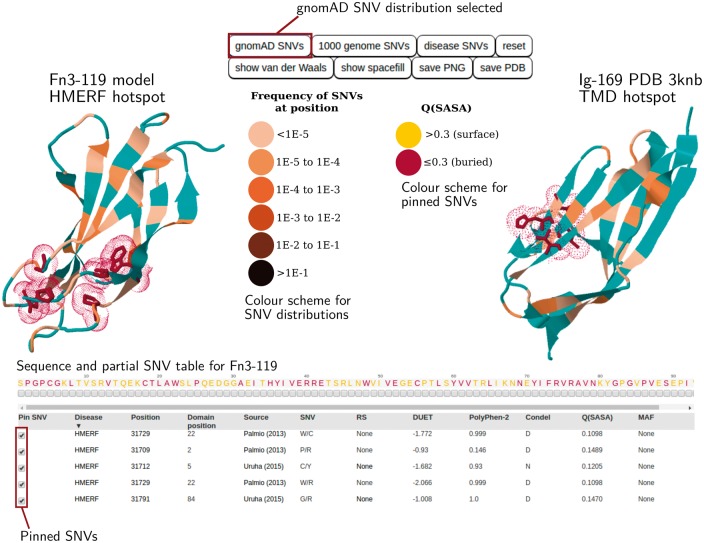
TITINdb user interface overview. The HMERF and TMD associated nsSNV hotspots are shown. Users can pin disease-associated nsSNVs from the SNV table onto domain structure and visualize these against the distribution of population nsSNVs (Gnomad or 1000 genomes). Pre-computed *in silico* analyses are shown in the SNV table (more information can be accessed by scrolling horizontally and vertically)

A potential application of TITINdb that involves investigating SNVs associated with specific diseases is shown in Figure 1 and further explored in section S2.1 of the Supplementary Materials. The facility to search by disease enables the detection of patterns or hotspots characteristic of variants associated with particular diseases. Two known nsSNV hotspots exist: one in domain Fn3-119 associated with hereditary myopathy with early respiratory failure (HMERF) (Pfeffer *et al.*, 2015) and one in Ig-169 associated with tibial muscular dystrophy, limb-girdle muscular dystrophy 2J (TMD/LGMD2J) (Chauveau *et al.*, 2014a; Hackman *et al.*, 2002; Savarese *et al.*, 2016).

TITINdb facilitates the visualization of nsSNVs associated with these diseases on structure (see Fig. 1); for both conditions nsSNVs can be observed to cluster in 3D space. In each case it can also be clearly seen that the distribution of disease associated nsSNVs on 3D structure is distinct from the distribution of population nsSNVs from the gnomAD database. Furthermore it becomes clear that all disease-associated nsSNVs discussed here are fairly buried (as indicated by a burgundy colour); therefore, it appears likely that they may disrupt protein stability. From the pre-calculated *in silico* analysis, it can be seen that all these disease associated nsSNVs are predicted to be destabilizing by DUET (Pires *et al.*, 2014a). TMD associated nsSNVs are also predicted, by mCSM (Pires *et al.*, 2014b), to disrupt the interaction between titin and obscurin, albeit by varying magnitudes; this has been validated experimentally (Fukuzawa *et al.*, 2008; Rudloff *et al.*, 2015). Interestingly the I35947N variant is predicted to have the least impact on the titin-obscurin interaction affinity (mCSM score -0.17 kcal/mol) out of all the TMD associated variants; this correlates with *in vitro* experimental observations where negligible differences have been found between this variant and wild-type titin (Fukuzawa *et al.*, 2008; Rudloff *et al.*, 2015). Additionally, the majority of HMERF associated nsSNVs are predicted to be deleterious by Condel (González-Pérez and López-Bigas, 2011), whereas only half the TMD associated nsSNVs are predicted to be deleterious. This highlights the need to take into consideration multiple sources of information, as provided by TITINdb, when predicting the potential impact of nsSNVs, and does not exclude experimental validation on a case-by-case basis.

Despite being a hotspot for HMERF associated nsSNVs, no experimental PDB structures or models are currently publicly available for the domain Fn3-119. Therefore, TITINdb has made possible the visualization of HMERF nsSNVs on structure and the *in silico* prediction of their impact at the molecular level. Multiple PDB structures exist for the domain Ig-169 (commonly referred to as M-10), to which TMD associated nsSNVs localize. Here, users can select which structure they wish to use to perform nsSNV visualization.

### 3.2 Investigating NGS nsSNV data

An application of TITINdb we believe to be particularly useful is the analysis of variants from NGS data. Specifically, the tool can be used in the prioritization of rare variants observed in disease cohorts for further experimental investigation. An example of such a variant is the P13979S titin N2B (isoform) nsSNV, which is published in the Supplementary Information associated with the article from Lopes *et al.* (2013), and further described in Section S2.2 of the Supplementary Materials. The nsSNV is found in 3/143 patients with HCM, leading to a cohort minor allele frequency (MAF) of between 0.01 and 0.02 (details on zygosity are not available).

A notable feature of TITINdb is the ability to search by different isoform positions. Tools such as ANNOVAR (Wang *et al.*, 2010) enable researchers to map variants from genomic to protein coordinates, however, depending on the protocol followed, variants may be mapped to different isoforms. For our nsSNV of interest, the N2B isoform coordinate is reported, thus the ‘search by position’ facility allows it to be mapped to both other major isoforms and the position within the affected domain. Additionally, it can be seen that the nsSNV localizes to residue position 5 of domain Fn3-55 and is present in all isoforms apart from the novex-3 isoform; therefore it is expressed in both cardiac and skeletal muscle.

TITINdb allows easy access to information concerning the nsSNV’s potential impact. Structurally, it can be seen that the affected residue has a Q(SASA) of 0.1 (this information is provided in the table on the nsSNV page), indicating that it is buried and that the nsSNV could potentially cause disease through destabilization of the underlying domain. It can also be seen, from predictions by the software SPPIDER (Porollo *et al.*, 2007), that the affected residue is not predicted to be involved in protein-protein interactions and thus is unlikely to cause disease through the disruption of these.

On comparison to known nsSNVs it can be seen that the nsSNV is present in both the 1000 genomes data and the gnomAD database with MAFs of 3.7036E-03 and 9.98403E-04. This indicates the variant is rare but present in a small proportion of nominally healthy individuals. From the MAF values it can be deduced that the variant is enriched in the HCM cohort (which we know has a MAF between 0.01 and 0.02). This suggests that the variant is either neutral, disease-causing with incomplete penetrance, recessive, or that a small number of nominally healthy individuals have undiagnosed HCM.

Structure (DUET)- and sequence (Condel)-based predictions of the impact of the nsSNV can be observed. The DUET score of −2.703 kcal/mol suggests the variant is highly destabilizing and supports the hypothesis derived earlier from the Q(SASA) that the variant could potentially lead to disease by disrupting the domain structure. Furthermore, it can be seen that the variant is also predicted to be deleterious by Condel.

As no experimental structures exist for the domain Fn3-55, 3D visualization and access to pre-computed structural analyses are made possible by the homology model provided as part of TITINdb. One salient feature is that, if nsSNVs are pinned on structure from the sequence, any related/identical nsSNVs rise to the top of the nsSNV table and become highlighted in either yellow (surface) or red (buried) according to their Q(SASA) (see Supplementary Figure S2); the pinned nsSNVs also follow this colour scheme.

The results indicate that, although the analysed nsSNV is highly likely to affect the domain structure, it is unclear whether this will contribute to the disease phenotype (primarily as the mutant titin may not be expressed *in vivo* in heterozygous cases).

Further information concerning applications of TITINdb can be found in the Supplementary Materials. In particular, it is hoped that the tool will enable clinicians to perform the information-based assessment of variants from patient data, and assist biologists in the prioritization of domain structures for biophysical characterization.

## Funding

This work has been supported by the British Heart Foundation [RE/13/2/30182, RG/15/8/31480 and CH/08/001] and the Biotechnology and Biological Sciences Research Council [BB/H018409/1 to FF].

*Conflict of Interest:* none declared.

## Supplementary Material

Supplementary Data
