# Supplementary material for: TITINdb—a computational tool to assess titin’s role as a disease gene
Source: Bioinformatics. 2017 Jul 4;33(21):3482–5. doi: 10.1093/bioinformatics/btx424 (PMC5860166; doi:10.1093/bioinformatics/btx424)
Supplement: Supplementary Data [file btx424_supplementary_information.pdf]

Supplementary Materials

S1 Application features

TITINdb enables users to:

- access pre-computed predictions of the impact of gnomAD, 1000 genomes and disease associated nsSNVs calculated using Duet (Pires et al., 2014b) and Condel (González-Pérez and López-Bigas, 2011)
- access pre-computed predictions of the impact of any possible single amino acid variants (SAVs) which localise to titin domain structures, calculated using mCSM (Pires et al., 2014a), and the impact of any possible nsSNVs calculated using Condel (González-Pérez and López-Bigas, 2011)
- access pre-computed predictions of the impact of any possible SAVs on protein-protein binding affinity calculated using mCSM (Pires et al., 2014a) (where experimental structures for titin domains in complex with interaction partners exist)
- visualise nsSNVs on structure and save images as PNG files
- visualise 3D nsSNV distributions on structure from the 1000 genomes project, gnomAD and disease associated nsSNVs
- download all nsSNV information for nsSNVs which localise to a titin Ig, Fn3 or kinase domains as a CSV file
- explore disease hotspots through the facility to "search by disease"
- access structural analysis, i.e. the Q(SASA) for each residue (computed using POPS (Cavallo et al., 2003)) and predictions of which residues take part in protein-protein interactions (computed using SPPIDER (Porollo et al., 2007))
- access Uniprot (The UniProt Consortium, 2017) functional site annotations, including residue modifications.
- download structures and models of titin Ig, Fn3 and kinase domains as PDB files
- assess model quality by using zDOPE score, per residue DOPE score plots and query-template alignments
- upload structures/models for nsSNV visualisation
- translate between isoform amino acid positions for all seven titin isoforms with RefSeq sequences (IC, N2AB, N2A, N2B, novex-1, novex-2, novex-3)

S2 Case studies

Mutations which result in amino acid changes can be broadly divided into two classes: those which are present in the population and which are either not phenotypically deleterious, or those which are causative of disease. Certain mutations may also be unclassified, and of uncertain effect. For titin this is frequently the case when nsSNVs are observed in disease cohorts but a causative link with the condition has not been established. In TITINdb we present analyses of nsSNVs which fall into the "classified" classes, to enable users to better understand the properties of such nsSNVs, and in order to facilitate the classification of currently unclassified nsSNVs; a graphical summary of this can be seen in Fig. S1. The two classes are, however, not mutually exclusive. Some disease associated nsSNVs may demonstrate incomplete penetrance or may be recessive (e.g. in compound heterozygosity (Evilä et al., 2014; Chauveau et al., 2014a)), or play a modifier role in disease. Conversely, it is possible that certain nsSNVs

may be misclassified due to nominally healthy individuals harbouring undiagnosed disease, or linkage disequilibrium existing between variants which are actually disease causing and those which are not. Indeed, it must be remembered that the gnomAD database, although not expected to be enriched in disease associated nsSNVs, does contain genetic information from disease cohorts, although individuals with severe paediatric disease have been filtered out (Lek et al., 2016). Nevertheless the gnomAD database is expected to contain rare recessive disease-causing TTN mutations similar to any gene, e.g. recessive autosomal CFTR mutations causing cystic fibrosis (frequency of heterozygous carriers between 1% and 3.5% depending on population (Strom et al., 2011)) or recessive X-linked DMD mutations causing Duchenne muscular dystrophy (carrier frequency 0.18% Mundy et al. (2016)). The main difference is that such recessive mutations are expected to be more numerous in titin due to the large size of the coding sequence (>100kb). *In silico* saturation mutagenesis has also been carried out to enable users to predict the impact of novel nsSNVs.

In the following case studies, we show how TITINdb allows the exploration of disease associated nsSNVs, and how the application can be used to facilitate the classification of new nsSNVs.

S2.1 Investigating disease associated nsSNVs

TITINdb allows the user to search by disease; currently 12 myopathies have associated titin variants. This enables the detection of patterns or hotspots characteristic of variants associated with particular diseases. Two known nsSNV hotspots have been investigated in the main text: one in domain Fn3-119 associated with hereditary myopathy with early respiratory failure (HMERF) (Palmio et al., 2014; Pfeffer et al., 2012; Ohlsson et al., 2012; Toro et al., 2013; Izumi et al., 2013; Vasli et al., 2012; Uruha et al., 2015) and one in Ig-169 associated with tibial muscular dystrophy/limb-girdle muscular dystrophy 2J (TMD/LGMD2J) (Pollazzon et al., 2010; Van den Bergh et al., 2003; Hackman et al., 2002; Evila et al., 2016). Here, we present an additional example, which investigates nsSNVs associated with dilated cardiomyopathy (DCM). This disease is primarily associated with titin truncating variants (Schafer et al., 2016). There are, however, a number of nsSNVs which are also associated with this condition.

On searching for dilated cardiomyopathy (DCM) associated nsSNVs (Itoh-Satoh et al., 2002; Gerull et al., 2002; Herman et al., 2012; LIU et al., 2008; Roncarati et al., 2013; Matsumoto et al., 2005), it can be seen that these do not appear to form a distinct hotspot as observed for both TMD and HMERF, and, although two of the thirteen nsSNVs localise to the domain Ig-30, they are situated at opposite ends of the domain. It can be noted that these nsSNVs are both found in gnomAD and one is also found in the 1000 genomes project; indicating that these nsSNVs are unlikely to demonstrate complete penetrance. Upon visualisation on the available model of Ig-30, it can be seen that the affected residues are also both located on the surface of the protein, with the S4780N nsSNV not predicted to be destabilising. This suggests that pathogenicity could be a result of the disruption of protein-protein interactions. Indeed, from the SNV table it can be seen that both residues affected by these nsSNVs are predicted to take part in protein-protein interactions.

From this example and those in the main text, it can be seen that inferring titin disease nsSNV pathogenicity can be a complex task; however, even with the sparse association of titin nsSNVs with disease, emerging patterns/hotspots are already discernible and further work will aid in the classification of currently unclassified nsSNVs.

S2.2 Investigating NGS nsSNV data

Hypertrophic cardiomyopathy (HCM) is a clinically heterogeneous disease in which the walls of the heart become thickened, in particular the wall of the left ventricle (Pantazis et al., 2015). It is caused by cardiac sarcomere mutations with incomplete penetrance (Seidman and Seidman, 2011) and is one of the leading causes of sudden death in young adults, affecting approximately 1 in 500 individuals (Maron et al., 1995). A number of rare and unique missense variants have recently been found in a cohort of HCM patients (Lopes et al., 2013), however how many of these play a causative role in the disease is currently unclear. In the

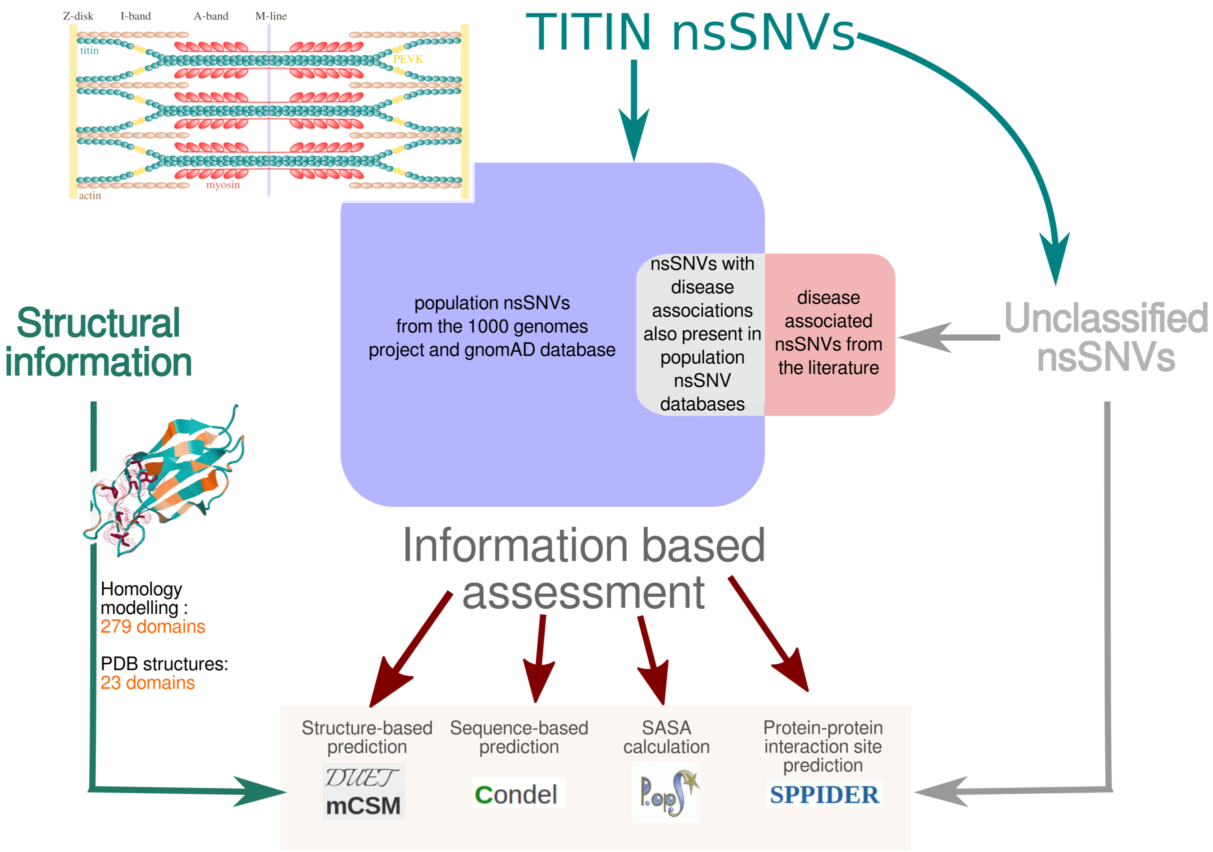

Figure S1: Summary showing how the information in TITINdb can be used to assess unclassified nsSNVs. This can be done in several ways, for example by comparing nsSNV properties to known SNVs, of which there are currently 51 disease associated nsSNVs and a larger number of population nsSNVs (19741 in the gnomAD database and 1982 in the 1000 genomes data). There is an overlap of 24 nsSNVs between disease associated nsSNVs and population nsSNVs (from the 1000 genomes project and gnomAD database); this is most likely due to the incomplete penetrance of some disease associated SNVs. Information based assessment can also contribute to the classification of unclassified SNVs through structure and sequence based predictions of their impact, and properties of their location on 3D structure.

main text we show one possible use of TITINdb through the analysis of the P13979S titin N2B nsSNV which is published in the supplementary information associated with the paper from Lopes et al. (2013). The visualisation of this particular nsSNV mapped to structure can be seen in Fig. S2.

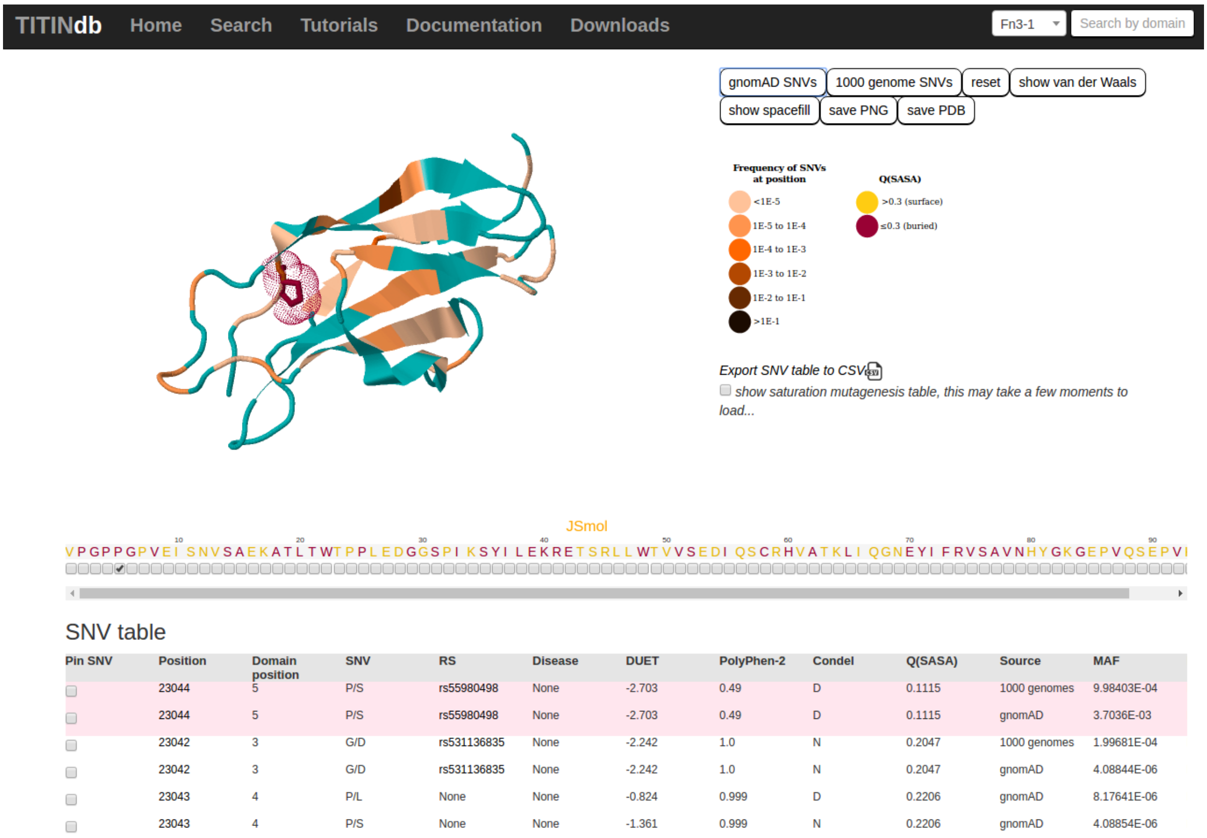

Figure S2: Visualisation of the P13979S titin N2B nsSNV on the Fn3-55 domain structure. The red colour indicates the nsSNV affects a buried residue. The nsSNV can be compared to the gnomAD distribution of nsSNVs (shown in orange).

S3 Methods

S3.1 Data

1000 genomes titin variant data was obtained in VCF format using the online data slicer (Auton et al., 2015). Variants were mapped to isoform and position using ANNOVAR (Wang et al., 2010). gnomAD variant data was obtained from the gnomAD web server (Lek et al., 2016) in Variant Dataset Format and titin nsSNVs were extracted using Hail (Ganna et al., 2016). Titin related disease nsSNVs were obtained from 'A rising titan: TTN review and mutation update' (Chauveau et al., 2014b). Disease nsSNVs reported in the literature discovered since the publication of Chauveau et al. (2014b) were queried for on PubMed using the terms "("titin"[All Fields]) AND ("snp"[All fields])","("titin"[All Fields]) AND ("mutation"[All fields])" and "("titin"[All Fields]) AND ("variant"[All fields])".

Data from the 1000 genomes project originates from 2504 nominally healthy individuals. The gnomAD database represents a much larger number of individuals (138632) and is aggregated from a number of studies, including the 1000 genomes project. It does not only include healthy individuals; however individuals with severe paediatric diseases have been filtered from the dataset. Therefore the gnomAD database is unlikely to be enriched in variants associated with severe diseases and is likely to be indicative of a population distribution of variants.

Titin functional site information was obtained from UniProt (The UniProt Consortium, 2017).

S3.2 Defining titin domain boundaries

HMMER (Finn et al., 2011) was used to scan the protein sequence of titin IC variant (NP_001254479.2), obtained from the RefSeq database (Pruitt et al., 2012) against Pfam seed libraries (Finn et al., 2014). Where hits overlapped the hit with the lowest E-value was accepted. When the lowest E-value hit for a region was greater than 0.0001 additional evidence was required to accept a hit, such as an existing experimental structure or high homology to other titin domains of the same type.

Sequences of titin domains defined in this way, including an extra 5 amino acids upstream and 16 amino acids downstream of the Pfam defined boundary, were aligned using T-coffee (Notredame et al., 2000) (separate alignments were created for titin Fn3 and Ig domain sequences).

Sequence logos were also created from these alignments using Weblogo (Notredame et al., 2000). Additionally sequence logos were created from the Pfam I-set and Fn3 seed alignments. It should be noted that all titin Ig domains are determined to be of the I-set type when defined by scanning against Pfam seed alignments.

Comparisons were made between sequence logos derived from aligned titin domain sequences and those derived from Pfam seed alignments. Where the two types of sequence logo differed substantially or the domain boundaries did not appear to be clearly defined by sequence conservation, the boundaries were mapped onto available experimental 3D structures and information from this mapping used to define titin domain boundaries.

S3.3 Mapping of titin isoforms

Stretcher (Myers and Miller, 1988) was used to align all titin isoforms to the IC variant. Isoform sequences were obtained from RefSeq (Pruitt et al., 2012). Positions were mapped according to these alignments.

S3.4 Modelling of titin domains

An automated homology modelling pipeline was set up. The pipeline takes a fasta file of domain sequences as input and uses only publicly available PDB structures as templates. The overall modelling process can be seen in Fig. S3A and a flow diagram detailing the template selection process is depicted in Fig. S3B. The template search, modelling and model assessment were performed using Modeller (Webb and Sali, 2016), the alignment of query and templates performed using 3DCoffee (O'Sullivan et al., 2004), and the overall pipeline produced using Python 2.7. Models were selected based on zDOPE score. zDOPE score is a normalised atomic distance-dependant statistical potential based on a sample of native structures (Shen and Sali, 2006). Lower zDOPE scores indicate better models with zDOPE scores below -1 indicating the distribution of atomic distances is similar to that in the sample of native structures.

The I-TASSER server (Zhang, 2008) was used to model Ig-112 as a satisfactory (negative) zDOPE score was not obtained using the homology modelling pipeline.

S3.5 Validation of models

The modelling pipeline described in section S3.3 was used to model all models with existing experimental structures. However so as to exclude the already solved structure from the templates, all hits with an identity >95% were excluded during template selection.

To validate the models these were compared to the representative experimental structures detailed in Table 1 in a similar manner to Sánchez and Sali (1998) as well as the Critical Assessment of Protein Structure Prediction experiments (Cozzetto et al., 2009). Sequence alignments between structures and models were calculated using Muscle (Edgar, 2004) and, structural superpositions guided by the sequence alignments as well as per residue RMSD calculations were performed using Theseus (Theobald and Wuttke, 2006).

S3.6 *In silico* assessment of the impact of nsSNVs

The *in silico* assessment of known nsSNVs occurring within Ig and Fn3 domains was performed using DUET (Pires et al., 2014b). This tool exploits structural information and is

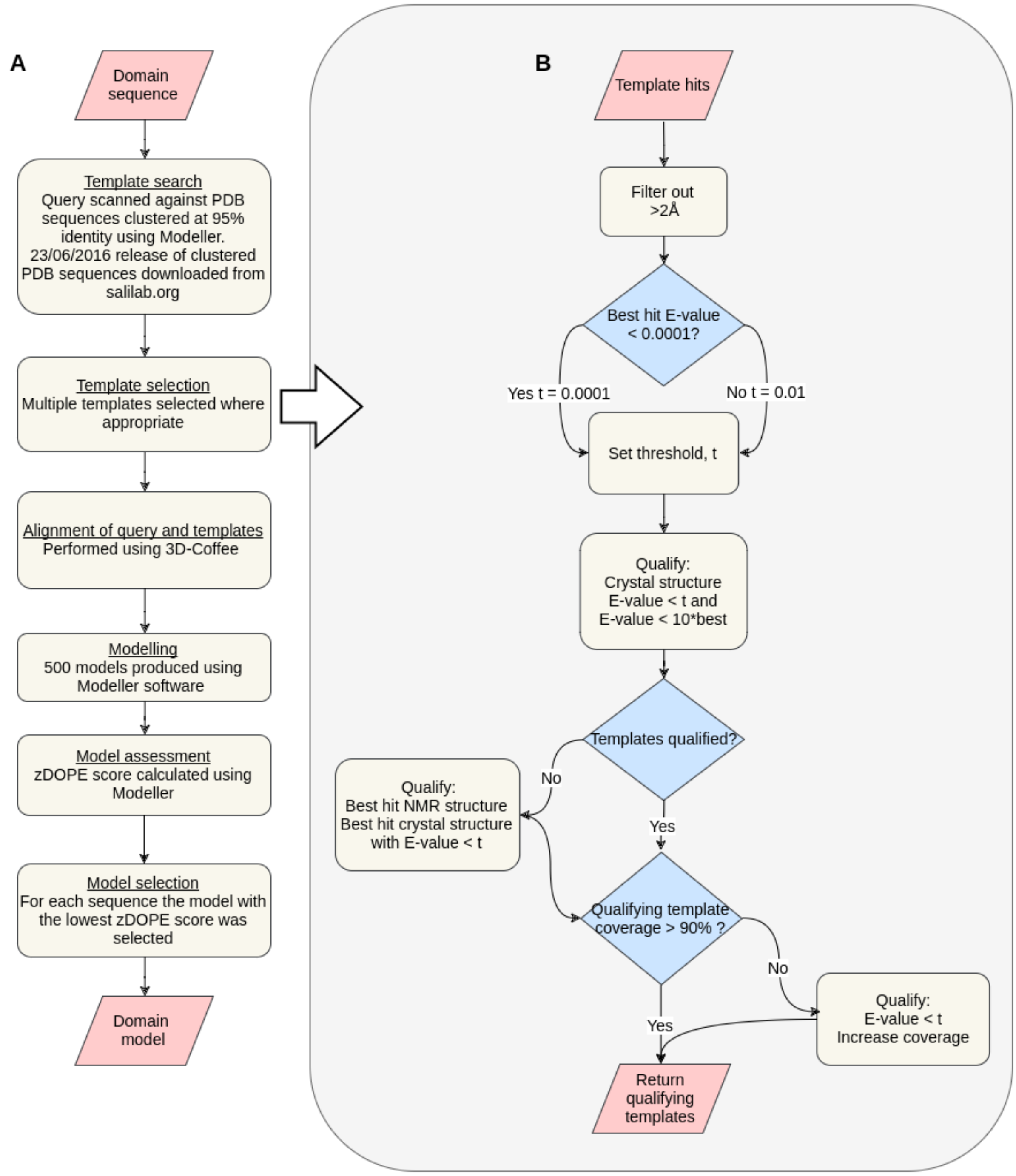

Figure S3: Flow-diagrams showing **A** an outline of the modelling pipeline and **B** template selection criteria. Qualifying templates refer to those templates which are selected for the modelling procedure.

based on a consensus of mCSM (Pires et al., 2014a), a graph-based method combined with machine learning to predict free energy changes resulting from single point mutations, and SDM

(Topham et al., 1997) which uses environment specific substitution tables. The prediction of impact for all possible SAVs which localise to domain structures was carried out using mCSM (Pires et al., 2014a); this algorithm was chosen as its speed enables the prediction to be carried out for the large number of such possible SAVs (492271). Where experimental structures were available these were used for the assessment. Where no experimental structures were available the model with the lowest zDOPE score was used. See table S1 for experimental structures used in the *in silico* assessment of nsSNVs. The impact of of nsSNVs on protein-protein binding affinity was also predicted using mCSM (Pires et al., 2014a) where experimental structures of titin domains in complex with binary interaction partners exist. See table S2 for structures used in the assessment of the impact of nsSNVs on protein-protein binding affinity.

Table S1: PDB structures used for the structural investigation of nsSNVs.

domain	PDB ID	method	resolution/ Å
Ig-1	2a38	X-RAY	2.00
Ig-2	2a38	X-RAY	2.00
Ig-10	1g1c	X-RAY	2.10
Ig-18	5jdd	X-RAY	1.53
Ig-19	5jdd	X-RAY	1.53
Ig-20	5jdd	X-RAY	1.53
Ig-84	5j0e	X-RAY	2.00
Ig-94	1waa	X-RAY	1.80
Ig-156	3lcy	X-RAY	2.50
Ig-157	3lcy	X-RAY	2.50
Ig-158	2j8h	X-RAY	1.99
Ig-159	2j8h	X-RAY	1.99
Ig-160	2bk8	X-RAY	1.69
Ig-163	3qp3	X-RAY	2.00
Ig-164	1tnn	NMR	NA
Ig-166	3puc	X-RAY	0.96
Ig-169	3knb	X-RAY	1.40
Fn3-3	4o00	X-RAY	1.85
Fn3-62	1bpv	NMR	NA
Fn3-66	3lpw	X-RAY	1.65
Fn3-67	3lpw	X-RAY	1.65
Fn3-132	2nzi	X-RAY	2.90‘

Assessment of all nsSNVs was performed using the method Condel (González-Pérez and López-Bigas, 2011). This method is purely sequence based and uses the weighted average of the normalised scores of 5 methods: Log R Pfam E-value, MAPP, Mutation Assessor, Polyphen2 and Sift (González-Pérez and López-Bigas, 2011).

S3.7 Definition of structural elements

Interface and core regions were defined using POPS (Cavallo et al., 2003). Residues with a Q(SASA) (quotient solvent accessible surface area) > 0.3 were defined as being surface residues and those with a Q(SASA) ≤ 0.3 defined as core residues. Here Q(SASA) is defined as the quotient of the SASA (solvent accessible surface area) and Surf (surface area of the isolated residue).

Putative PPI interface regions were predicted using SPPIDER II (Porollo et al., 2007) with a balanced trade-off between sensitivity and specificity (SPPIDER estimates this based on a control data set of 149 protein chains with no sequence homology), using representative structures (see Table 1).

S3.8 Creation of a titin database

A database was set up to integrate titin structural, variant and disease information. The database was created using SQLite and DJANGO. nsSNVs from the 1000 genomes project, ExAC and the paper ‘A rising Titin: TTN review and mutation update’ (Chauveau et al., 2014b), as well as information concerning structures modelled by the pipeline and PDB structures were loaded to the database.

S3.9 Web server implementation

TITINdb is a python-based application implemented using the DJANGO framework. JSmol is used to visualise protein structures. It is hosted on an Apache2.2.15 web server.

Table S2: PDB structures used for the investigation of the impact of nsSNVs on protein-protein binding affinity.

domain	PDB ID	method	resolution / Å	interaction partner
Ig-1	1ya5	X-RAY	2.44	TCAP
Ig-2	1ya5	X-RAY	2.44	TCAP
Ig-169	3knb	X-RAY	1.40	OBSL1
Ig-2	4c4k	X-RAY	1.95	TCAP

S4 Results

S4.1 Comparison of the calculated properties of categorised nsSNVs/SAVs

A comparison of the calculated properties of categorised nsSNVs/SAVs which feature in TITINdb can be seen in Fig. S4. The top left plot compares the log transformed distribution of gnomAD frequencies for nsSNVs found in the 1000 genomes project with the log transformed distribution of gnomAD frequencies for nsSNVs associated with disease (a small pseudocount of 3.6E-6 has been added to each frequency to allow for log transformation). It can be seen that although the two subsets are significantly different the MAF values show a large overlap. Here the caveat must be taken that data from 1851 individuals from the 1000 genomes project is a subset of the gnomAD data set. However, as data from the 1000 genomes project only accounts for 1.3% of the data in gnomAD it is considered unlikely that this will have a large impact on the MAF values observed in gnomAD. These results suggest that not all pathogenic variants can be distinguished from non-disease causing variants by differences in MAF.

The top right plot shows the distributions of Condel scores for nsSNVs from the 1000 genomes project, the gnomAD database, saturation mutagenesis, and disease associated nsSNVs. The disease associated nsSNVs are predicted to be significantly more deleterious than nsSNVs the other subsets. nsSNVs from saturation mutagenesis are predicted to be significantly more deleterious than those from the 1000 genomes project and gnomAD database (from Fig. S4 it is clear the difference is very small and significance reached due to the large size of the dataset), however significantly less deleterious than disease associated variants.

In the bottom left plot it can be seen that disease associated nsSNVs localise to residues with a significantly lower Q(SASA) than nsSNVs and SAVs from all other subsets. SAVs from saturation mutagenesis localise to residues with a significantly lower Q(SASA) than variants from the 1000 genomes data or ExAC database, however it is clear that the difference is very small and significance reached due to the large size of the dataset.

The bottom right plot shows the distribution of mCSM scores (predicted impact on stability in kcal/mol, negative values indicate destabilisation) for variants from each subset of the data. Here it can be seen that disease associated nsSNVs are predicted to be significantly more destabilising than nsSNVs from the 1000 genomes project, the gnomAD database and SAVs from saturation mutagenesis.

Please note that saturation mutagenesis has explored all variants (SAVs) which can be achieved by a single amino acid change through mCSM and POPs, however only those variants (nsSNVs) which can be achieved by a single nucleotide change through Condel.

S4.2 Titin domain boundaries

We find titin has 169 Ig domains in contrast to the previously reported 152 (Chauveau et al., 2014b). This discrepancy arises from both the previous use of shorter isoforms to define domains rather than the reference IC isoform, and the failure to integrate available experimental evidence. Domain Ig-94 is identified with an E-value of 0.0079 which is higher than the set threshold of 0.0001, but is validated by the experimental PDB structures (1WAA, 1TIU and 1TIT) which exist of this

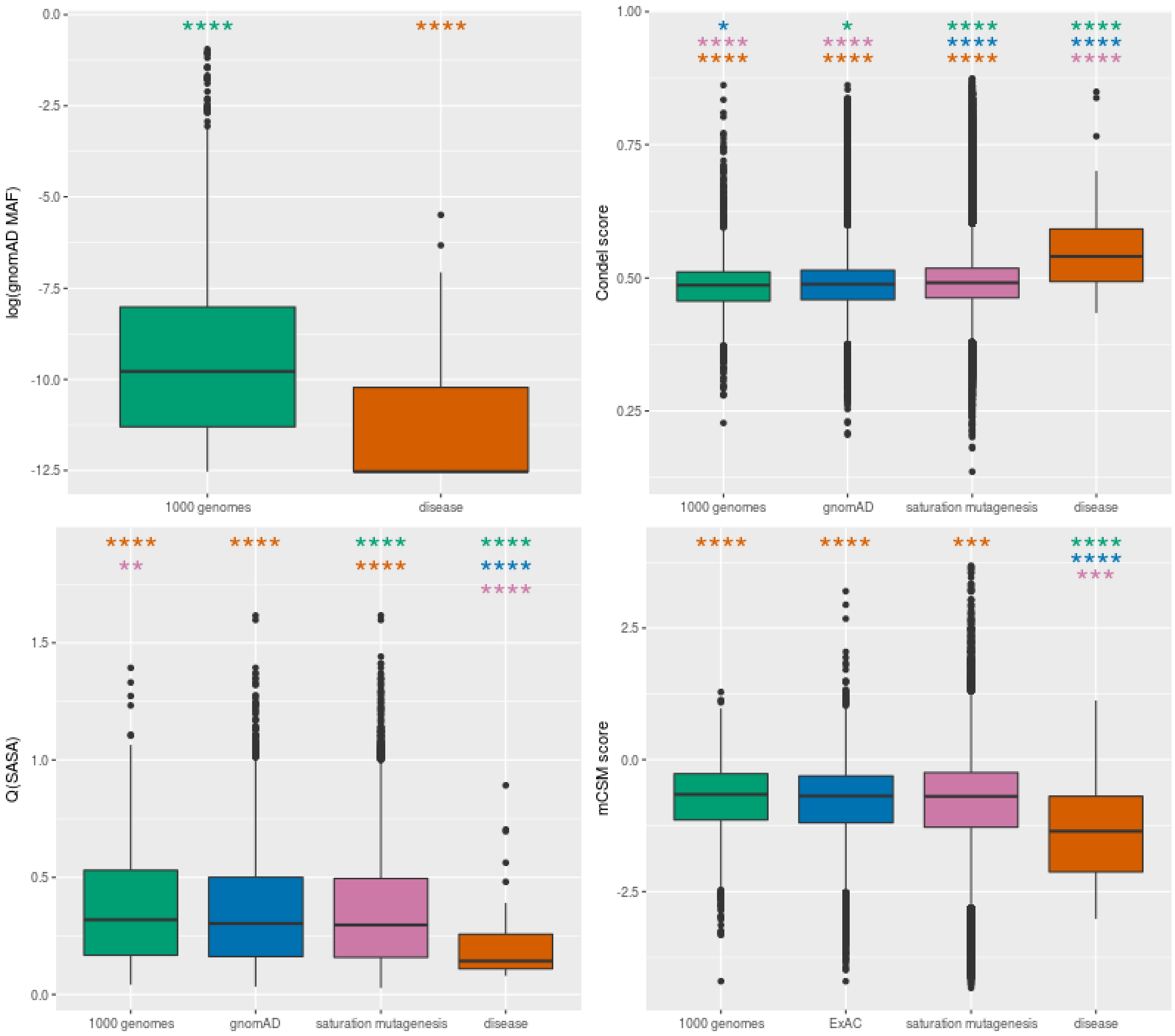

Figure S4: A comparison of the properties of titin nsSNVs from the 1000 genomes project and gnomAD database with disease associated nsSNVs and nsSNVs/SAVs from saturation mutagenesis. It can be seen that disease associated nsSNVs are predicted to be both significantly more destabilising and significantly more deleterious than nsSNVs/SAVs from the other subsets. It can also be seen that disease associated SNVs localise to residues with a significantly lower Q(SASA). Significance calculated using pairwise Mann-Whitney tests with Bonferroni correction (*p<0.05; **p<0.01; ***p<0.001; ****p<0.0001).

domain. Ig-88, Ig-89, Ig-90 and Ig-98 were also identified with E-values higher than the threshold (0.00079, 0.00026, 0.0022 and 0.0005); however when the titin sequence was scanned using an HMM created from an alignment of all (165) other titin Ig domains, these domains were identified with significantly low E-values (5.9E-11, 1.2E-12, 2.9E-10,7.4E-12). The mapping of all titin isoform protein sequence positions to the reference IC isoform has enabled the instigation of a consistent naming scheme for domains in which Ig domains are sequentially numbered from 1 to 169 and Fn3 domains numbered sequentially from 1 to 132.

Sequences logos (see Fig. S5) showing aligned titin Fn3 sequences, differ substantially from such logos depicting Pfam seed alignments; particularly

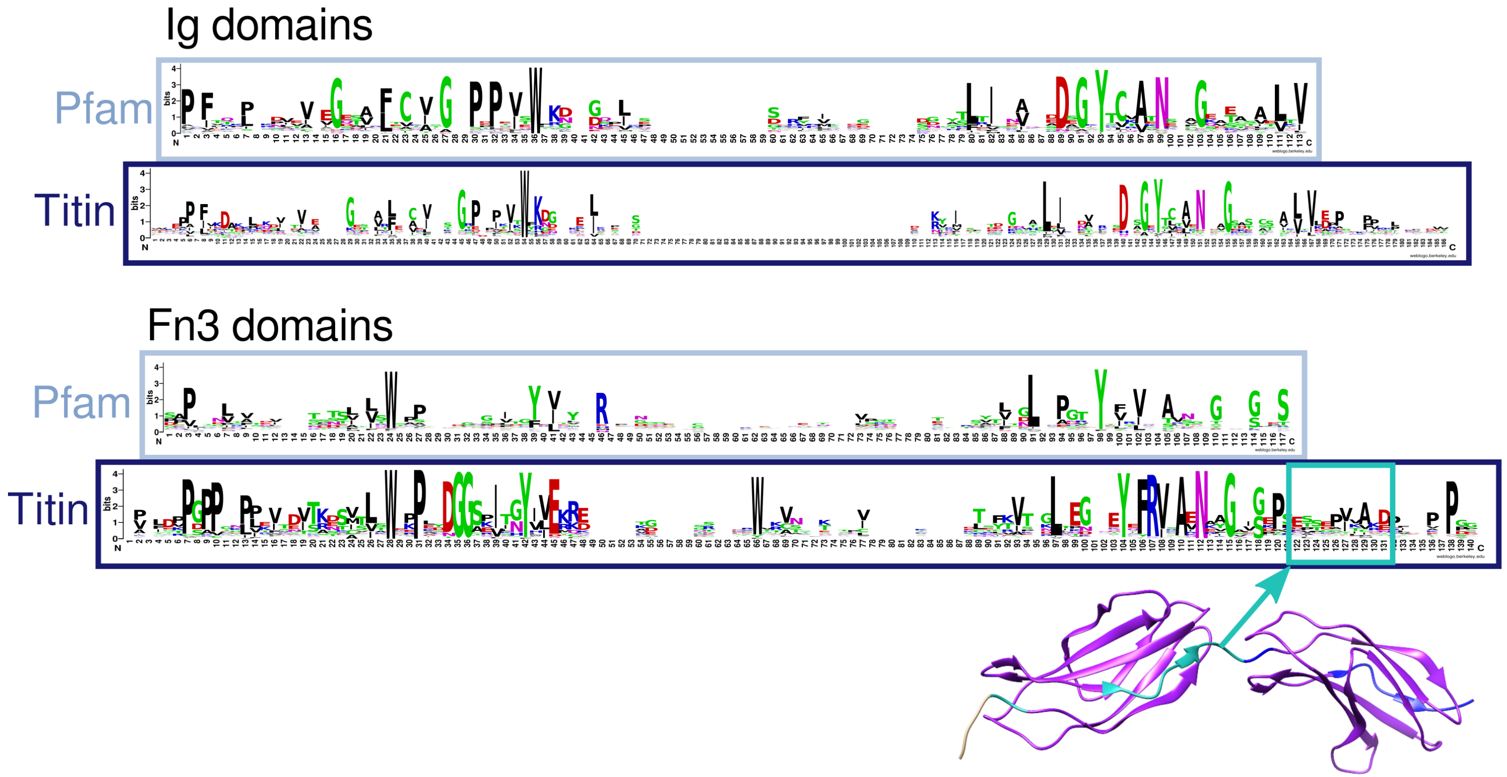

Figure S5: Sequence logos showing aligned titin domains and Pfam seed alignments for Ig and Fn3 domains. The Pfam Fn3 domain definition can be seen mapped onto the structure 3LPW in purple with structure absent from the Pfam definition in turquoise and blue.

towards the end of the sequence where the conservation drops of gradually. Therefore the correct boundaries do not appear to be clearly defined from sequence alone. When mapped onto available structures, for example in the case of the Fn3 dimer 3LPW (bottom Fig. S5), it becomes clear that the Pfam defined boundaries do not cover entire titin Fn3 domains. Due to this information it was decided the Pfam defined Fn3 domain boundaries were not accurately determined. Therefore Fn3 domains were initially identified using Pfam/HMMER and the sequences of these domains, including an extra 5 amino acids upstream and 16 amino acids downstream of the Pfam defined boundaries, were aligned using T-coffee. This alignment was cut using structural information from available titin Fn3 crystal structures, in particular 3LPW. An HMM was created from this alignment and titin scanned again using this HMM to redefine titin Fn3 domain boundaries.

S4.3 Modelling of titin Ig and Fn3 domains

A pipeline was set up as described in the methods section to perform the homology modelling of titin Fn3 and Ig domains. Fig. S6 shows the structural coverage of titin by experimental crystal/NMR structures from the PDB, existing models from the ModBase database (Pieper et al., 2009), and models produced by the TITINdb pipeline.

It can be seen in Fig. S6 that our pipeline has greatly increased both the structural coverage of domains and the quality of the coverage (lower zDOPE (Shen and Sali, 2006) scores indicate better structures with native structures expected to have a zDOPE score around -1). Here, for each domain sequentially along the length of titin, existing experimental structures are represented by purple diamonds and blue hexagons, ModBase models are represented by blue bars and models created by the TITINdb pipeline are represented by red bars. The closest identity each domain shares with an experimental PDB structure is annotated along the top x-axis.

Model validation was performed by modelling all domains for which experimental structures already exist, however excluding structures with >95% identity from template selection. The models were then compared to the solved structures for relevant domain as described in the methods section, in a similar manner to Sánchez and Sali (1998) and Cozzetto et al. (2009). Cumulative distribution plots for the RMSD (root-mean-square deviation) for the comparison between models and representative structures are shown in Fig. S7A (Ig domains) and Fig. S7C (Fn3 domains). It can be seen that a large proportion of modelled residues have RMSD values lower than 1Å indicating that the modelling has predicted the actual structure with a high degree of accuracy. For 82% of the models >60% of their residues fall within 1Å of the solved structure and for 65% of the models >70% of their residues fall within 1Å of the solved structure (see table S4.3). As experimental structures have only been solved for 5 titin Fn3 domains, summary statistics are perhaps less informative, however for 80% of these >70% of residues lie within 1Å of the solved crystal structures. For this small sample size the Fn3 models generally show less deviation in terms of alpha carbon (Cα) RMSD from the solved structures than the Ig models do. This is perhaps a result of the proportionally lower loop content of the majority of titin Fn3 domains when compared to titin Ig domains; as loop regions tend to be more flexible and less conserved these tend to result in larger RMSD values.

In the majority of cases those residues with the highest RMSDs when compared to solved structures localise to loops; as discussed this is unsurprising due to the inherently greater flexibility of loop regions. The exception to this is the model for Ig-19, shown aligned to the crystal structure 5JDD in Fig. S7A-I, which, from the cumulative RMSD distribution appears to have been modelled with the least success. On closer inspection it becomes apparent that the majority of the backbone aligns closely to the crystal structure however the loop prior to the C-terminus is not as tight as in the crystal structure which results in misplacement of the final β sheet. On observation of the alignment between the query and template sequences (see Fig. S8) it can be seen that the quality of the alignment drops for the portion of the sequence which corresponds to the C-terminal β sheet which perhaps explains the poor accuracy of modelling this region. The other immunoglobulin model which appears less accurate judging by the RMSD values is Ig-164, shown aligned to the NMR

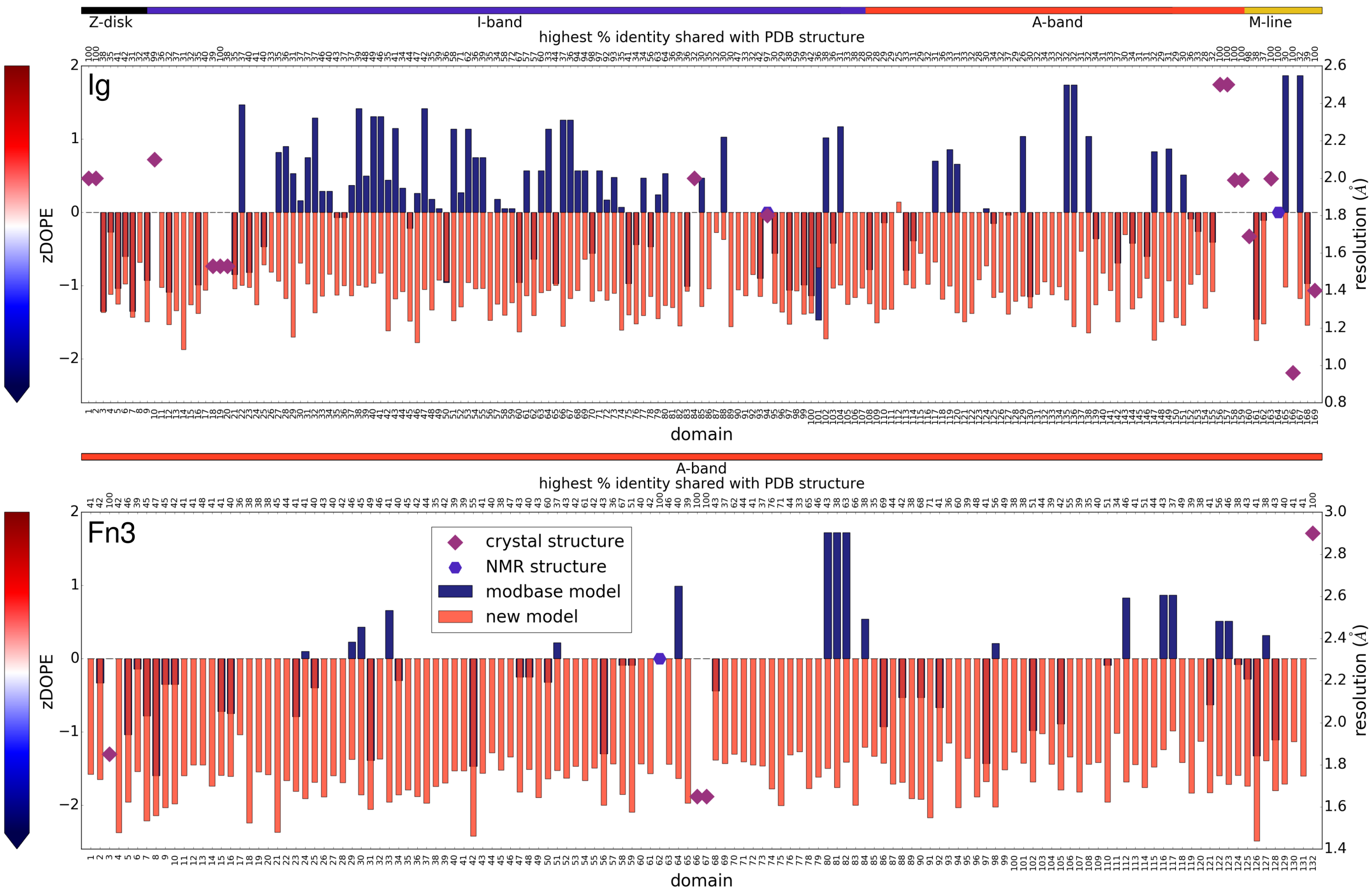

Figure S6: Structural coverage of titin Ig and Fn3 domains by models, crystal and NMR structures. New models refer to those produced during this project. Lower zDOPE scores indicate better model quality. The highest percentage identity shared with a homologous crystal structure (at the time of modelling) can be seen along the top x-axis for each domain.

structure 1TNN in Fig. S7A-II . Here, as expected, the beta sheet core regions show close alignment to the solved NMR structure 1TNN, however the loop regions show large differences, in particular a short helical stretch can be seen in the loop between beta sheets E and F in the model which is not present in the structure. Interestingly, the model was built using only crystal structures as templates, and all the crystal structures available for titin Ig domains show such a short helical segment in their analogous loops; however none of the NMR structures demonstrate this property. Therefore it is likely that the helical structure does not form in solution due to competition with the surrounding solvent for hydrogen bond formation with partially exposed residues. This indicates that the higher RMSD values observed in the loop regions for this domain may be an indicator of conformational differences caused by the distinct environments in which the structures have been solved, rather than poor model quality.

Table S3: Percentage of domains for which models have a particular percentage of residues within 1Å of the solved experimental structure after structural superposition. The analysis was performed using 17 domains for Ig domains and 5 domains for Fn3 domains (see Table S1 for domains and experimental structures used for validation).

% residues $<$1Å RMSD from representative structure	% Ig domains
10	100
20	100
30	100
40	100
50	94
60	82
70	65
80	35
90	12

% residues $<$1Å RMSD from representative structure	% Fn3 domains
10	100
20	100
30	100
40	100
50	100
60	80
70	80
80	60
90	0

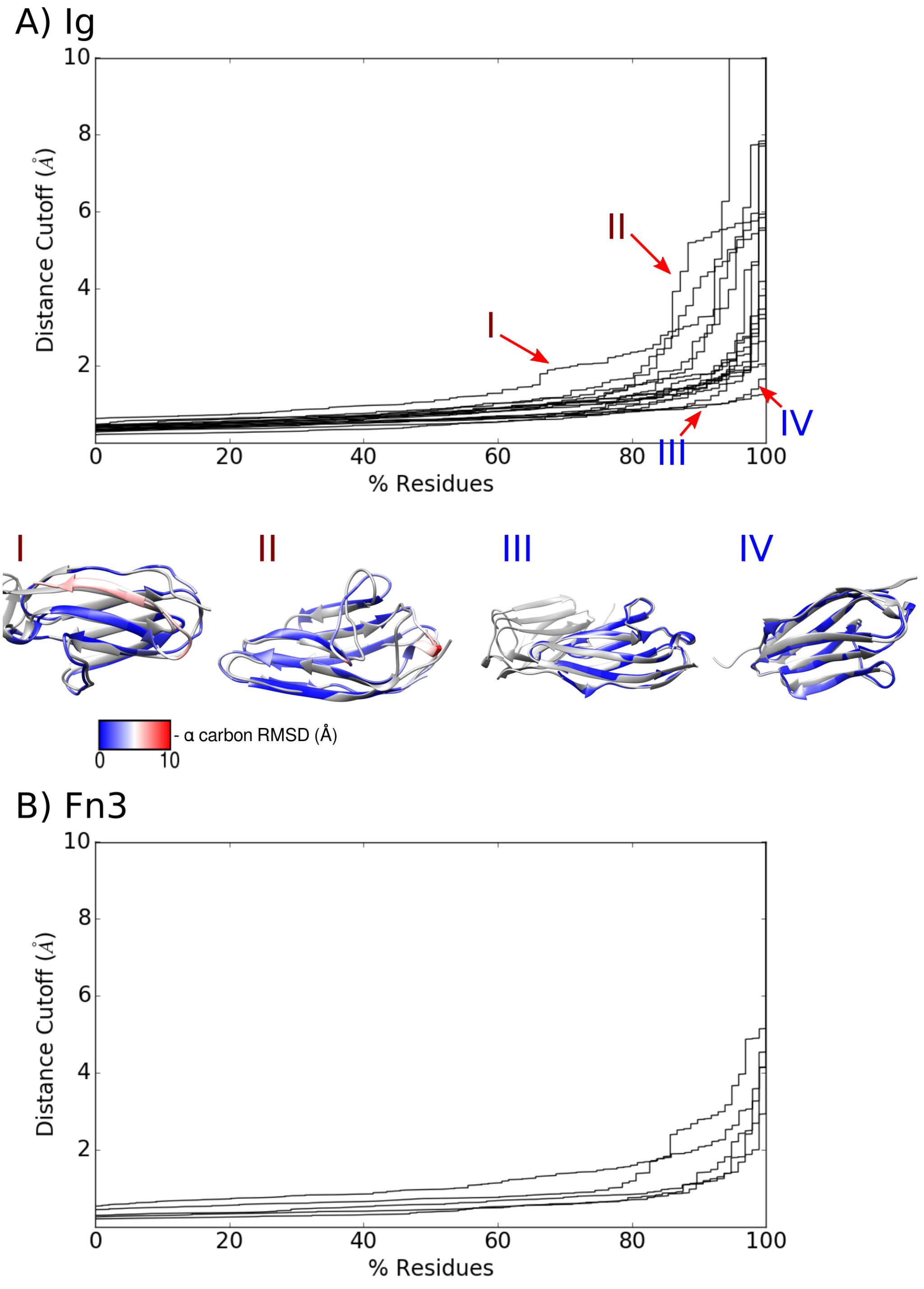

Figure S7: Cumulative RMSD plots for models aligned to representative structures **A** for Ig domains **B** for Fn3 domains. Alignments of Ig models with representative structures coloured by RMSD can be seen for two of the least successful cases (**I** Ig-19 aligned to 5JDD and **II** Ig-164 aligned to 1TNN) and two of the most successful cases (**III** Ig-2 aligned to 2A38 and **IV** Ig-163 aligned to 3QP3). A similar method of model assessment has been used by Sánchez and Sali (1998) and Cozzetto et al. (2009)

```
T-COFFEE, Version_11.00.8cbe486 2014-08-12 22:05:29 - Revision 8cbe486 - Build 477
   Cedric Notredame
   CPU TIME:0 sec.
   SCORE=938
   *
    BAD AVG GOOD
   *
   Ig-19     :   91
   2yuzA     :   91
   2dltA     :   91
   1x44A     :   91
   cons      :   93

   Ig-19     ---LHITKTMKNIEVPETKTASFECEVSHFNVPSMWLKNGVEIEM
   2yuzA     SSGLKILTPLTDQTVNLGKEICLKCEISE-NIPGKWTKNGLPVQE
   2dltA     SGQLEVLQDIADLTVKAAEQAVFKCEVSDEKVTGKWYKNGVEVRP
   1x44A     SSGIMVTKQLEDTTAYCGERVELECEVSEDDANVKWFKNGEEIIP

   cons         : :   : :  .   :   ::**:*. .    * ***  :  


   Ig-19     --SEKFKIVVQGKLHQLIIMNTSTEDSAEYTFVCGNDQVSATLTV
   2yuzA     --SDRLKVVQKGRIHKLVIANALTEDEGDYVFAPDAYNVTLPAKV
   2dltA     --SKRITISHVGRFHKLVIDDVRPEDEGDYTFVPDGYALSLSAKL
   1x44A     GPKSRYRIRVEGKKHILIIEGATKADAAEYSVMTTGGQSSAKLSV

   cons        ..:  :   *: *:* ..   * .:* .       :   .:


   Ig-19     T---
   2yuzA     HVIS
   2dltA     NFLE
   1x44A     DLKS

   cons      
```

Figure S8: Alignment of query sequence and templates for the domain Ig-19, obtained using T-coffee (Notredame et al., 2000).

References

A. Auton et al. A global reference for human genetic variation. *Nature*, 526(7571):68–74, 2015. doi: 10.1038/nature15393.

L. Cavallo et al. POPS: A fast algorithm for solvent accessible surface areas at atomic and residue level. *Nucleic Acids Res*, 31(13):3364–6, 2003.

C. Chauveau et al. Recessive TTN truncating mutations define novel forms of core myopathy with heart disease. *Hum Mol Genet*, 23(4): 980–91, 2014a. doi: 10.1093/hmg/ddt494.

C. Chauveau et al. A rising titan: TTN review and mutation update. *Hum Mutat*, 35(9):1046–59, 2014b. doi: 10.1002/humu.22611.

D. Cozzetto et al. Evaluation of template-based models in CASP8 with standard measures. *Proteins*, 77 Suppl 9:18–28, 2009. doi: 10.1002/prot.22561.

R.C. Edgar. MUSCLE: a multiple sequence alignment method with reduced time and space complexity. *BMC Bioinformatics*, 5:113, 2004. doi: 10.1186/1471-2105-5-113.

A. Evilä et al. Atypical phenotypes in titinopathies explained by second titin mutations. *Ann Neurol*, 75(2):230–40, 2014. doi: 10.1002/ana.24102.

A. Evila et al. Targeted Next-Generation Sequencing Reveals Novel TTN Mutations Causing Recessive Distal Titinopathy. *Mol. Neurobiol.*, Oct 2016. doi: 10.1007/s12035-016-0242-3.

R.D. Finn et al. HMMER web server: interactive sequence similarity searching. *Nucleic Acids Res*, 39(Web Server issue):W29–37, 2011. doi: 10.1093/nar/gkr367.

R.D. Finn et al. Pfam: the protein families database. *Nucleic Acids Res*, 42(Database issue): D222–30, 2014. doi: 10.1093/nar/gkt1223.

A. Ganna et al. Ultra-rare disruptive and damaging mutations influence educational attainment in the general population. *Nat. Neurosci.*, 19(12): 1563–1565, Dec 2016. doi: 10.1038/nn.4404.

B. Gerull et al. Mutations of TTN, encoding the giant muscle filament titin, cause familial dilated cardiomyopathy. *Nat Genet*, 30(2):201–4, 2002. doi: 10.1038/ng815.

A. González-Pérez and N. López-Bigas. Improving the assessment of the outcome of nonsynonymous SNVs with a consensus deleteriousness score, Condel. *Am J Hum Genet*, 88(4):440–9, 2011. doi: 10.1016/j.ajhg.2011.03.004.

P. Hackman et al. Tibial muscular dystrophy is a titinopathy caused by mutations in TTN, the gene encoding the giant skeletal-muscle protein titin. *Am J Hum Genet*, 71(3):492–500, 2002. doi: 10.1086/342380.

D.S. Herman et al. Truncations of titin causing dilated cardiomyopathy. *N Engl J Med*, 366(7): 619–28, 2012. doi: 10.1056/NEJMoa1110186.

M. Itoh-Satoh et al. Titin mutations as the molecular basis for dilated cardiomyopathy. *Biochem Biophys Res Commun*, 291(2):385–93, 2002. doi: 10.1006/bbrc.2002.6448.

R. Izumi et al. Exome sequencing identifies a novel TTN mutation in a family with hereditary myopathy with early respiratory failure. *J Hum Genet*, 58(5):259–66, 2013. doi: 10.1038/jhg.2013.9.

M. Lek et al. Analysis of protein-coding genetic variation in 60,706 humans. *Nature*, 536(7616): 285–91, 2016. doi: 10.1038/nature19057.

X. LIU et al. [Titin gene mutations in Chinese patients with dilated cardiomyopathy]. *Zhonghua Xin Xue Guan Bing Za Zhi*, 36(12):1066–9, 2008.

L.R. Lopes et al. Genetic complexity in hypertrophic cardiomyopathy revealed by high-throughput sequencing. *J Med Genet*, 50(4):228–39, 2013. doi: 10.1136/jmedgenet-2012-101270.

B.J. Maron et al. Prevalence of hypertrophic cardiomyopathy in a general population of young adults. Echocardiographic analysis of 4111 subjects in the CARDIA Study. Coronary Artery Risk Development in (Young) Adults. *Circulation*, 92(4):785–9, 1995.

Y. Matsumoto et al. Functional analysis of titin/connectin N2-B mutations found in cardiomyopathy. *J Muscle Res Cell Motil*, 26(6-8):367–74, 2005. doi: 10.1007/s10974-005-9018-5.

S. Mundy et al. Duchenne/becker muscular dystrophy: advances in reproductive testing options. *Fertility and Sterility*, 106(3):e372, 2016. doi: 10.1016/j.fertnstert.2016.07.1058.

E.W. Myers and W. Miller. Optimal alignments in linear space. *Comput Appl Biosci*, 4(1):11–7, 1988.

C. Notredame et al. T-Coffee: A novel method for fast and accurate multiple sequence alignment. *J Mol Biol*, 302(1):205–17, 2000. doi: 10.1006/jmbi.2000.4042.

M. Ohlsson et al. Hereditary myopathy with early respiratory failure associated with a mutation in A-band titin. *Brain*, 135(Pt 6):1682–94, 2012. doi: 10.1093/brain/aws103.

O. O'Sullivan et al. 3DCoffee: combining protein sequences and structures within multiple sequence alignments. *J Mol Biol*, 340(2):385–95, 2004. doi: 10.1016/j.jmb.2004.04.058.

J. Palmio et al. Hereditary myopathy with early respiratory failure: occurrence in various populations. *J Neurol Neurosurg Psychiatry*, 85(3):345–53, 2014. doi: 10.1136/jnnp-2013-304965.

A. Pantazis et al. Diagnosis and management of hypertrophic cardiomyopathy. *Echo Res Pract*, 2(1):R45–53, 2015. doi: 10.1530/ERP-15-0007.

G. Pfeffer et al. Titin mutation segregates with hereditary myopathy with early respiratory failure. *Brain*, 135(Pt 6):1695–713, 2012. doi: 10.1093/brain/aws102.

U. Pieper et al. MODBASE, a database of annotated comparative protein structure models and associated resources. *Nucleic Acids Res*, 37(Database issue):D347–54, 2009. doi: 10.1093/nar/gkn791.

D.E. Pires et al. mCSM: predicting the effects of mutations in proteins using graph-based signatures. *Bioinformatics*, 30(3):335–42, 2014a. doi: 10.1093/bioinformatics/btt691.

D.E. Pires et al. DUET: a server for predicting effects of mutations on protein stability using an integrated computational approach. *Nucleic Acids Res*, 42(Web Server issue):W314–9, 2014b. doi: 10.1093/nar/gku411.

M. Pollazzon et al. The first Italian family with tibial muscular dystrophy caused by a novel titin mutation. *J Neurol*, 257(4):575–9, 2010. doi: 10.1007/s00415-009-5372-3.

A. Porollo et al. Prediction-based fingerprints of protein-protein interactions. *Proteins*, 66(3): 630–45, 2007. doi: 10.1002/prot.21248.

K.D. Pruitt et al. NCBI Reference Sequences (RefSeq): current status, new features and genome annotation policy. *Nucleic Acids Res*, 40(Database issue):D130–5, 2012. doi: 10.1093/nar/gkr1079.

R. Roncarati et al. Doubly heterozygous LMNA and TTN mutations revealed by exome sequencing in a severe form of dilated cardiomyopathy. *Eur J Hum Genet*, 21(10): 1105–11, 2013. doi: 10.1038/ejhg.2013.16.

R. Sánchez and A. Sali. Large-scale protein structure modeling of the Saccharomyces cerevisiae genome. *Proc Natl Acad Sci U S A*, 95(23):13597–602, 1998.

S. Schafer et al. Titin-truncating variants affect heart function in disease cohorts and the general population. *Nat. Genet.*, Nov 2016. doi: 10.1038/ng.3719.

C.E. Seidman and J.G. Seidman. Identifying sarcomere gene mutations in hypertrophic cardiomyopathy: a personal history. *Circ Res*, 108(6):743–50, 2011. doi: 10.1161/CIRCRESAHA.110.223834.

M.Y. Shen and A. Sali. Statistical potential for assessment and prediction of protein structures. *Protein Sci*, 15(11):2507–24, 2006. doi: 10.1110/ps.062416606.

C. M. Strom et al. Cystic fibrosis testing 8 years on: lessons learned from carrier screening and sequencing analysis. *Genet. Med.*, 13(2):166–172, Feb 2011. doi: 10.1097/GIM.0b013e3181fa24c4.

The UniProt Consortium. UniProt: the universal protein knowledgebase. *Nucleic Acids Res.*, 45(D1):D158–D169, Jan 2017. doi: 10.1093/nar/gkw1099.

D.L. Theobald and D.S. Wuttke. THESEUS: maximum likelihood superpositioning and analysis of macromolecular structures. *Bioinformatics*, 22(17):2171–2, 2006. doi: 10.1093/bioinformatics/btl332.

C.M. Topham et al. Prediction of the stability of protein mutants based on structural environment-dependent amino acid substitution and propensity tables. *Protein Eng*, 10(1):7–21, 1997.

C. Toro et al. Exome sequencing identifies titin mutations causing hereditary myopathy with early respiratory failure (HMERF) in families of diverse ethnic origins. *BMC Neurol*, 13:29, 2013. doi: 10.1186/1471-2377-13-29.

A. Uruha et al. Necklace cytoplasmic bodies in hereditary myopathy with early respiratory failure. *J Neurol Neurosurg Psychiatry*, 86(5): 483–9, 2015. doi: 10.1136/jnnp-2014-309009.

P.Y. Van den Bergh et al. Tibial muscular dystrophy in a Belgian family. *Ann Neurol*, 54 (2):248–51, 2003. doi: 10.1002/ana.10647.

N. Vasli et al. Next generation sequencing for molecular diagnosis of neuromuscular diseases. *Acta Neuropathol*, 124(2):273–83, 2012. doi: 10.1007/s00401-012-0982-8.

K. Wang et al. ANNOVAR: functional annotation of genetic variants from high-throughput sequencing data. *Nucleic Acids Res*, 38(16): e164, 2010. doi: 10.1093/nar/gkq603.

B. Webb and A. Sali. Comparative Protein Structure Modeling Using MODELLER. *Curr Protoc Protein Sci*, 86:2.9.1–2.9.37, 2016. doi: 10.1002/cpps.20.

Y. Zhang. I-TASSER server for protein 3D structure prediction. *BMC Bioinformatics*, 9:40, 2008. doi: 10.1186/1471-2105-9-40.
